# Unipolar and Bipolar Depression Detection and Classification Based on Actigraphic Registration of Motor Activity Using Machine Learning and Uniform Manifold Approximation and Projection Methods

**DOI:** 10.3390/diagnostics13142323

**Published:** 2023-07-10

**Authors:** Mohammed Zakariah, Yousef Ajami Alotaibi

**Affiliations:** 1 Department of Computer Science, College of Computer and Information Sciences, King Saud University, Riyadh P.O. Box 11442, Saudi Arabia; mzakariah@ksu.edu.sa; 2 Department of Computer Engineering, College of Computer and Information Sciences, King Saud University, Riyadh P.O. Box 11451, Saudi Arabia

**Keywords:** depression, unipolar detection, bipolar disorder, classification-based, motor activity, machine-learning, UMAP method, actigraphic registration

## Abstract

Modern technology frequently uses wearable sensors to monitor many aspects of human behavior. Since continuous records of heart rate and activity levels are typically gathered, the data generated by these devices have a lot of promise beyond counting the number of daily steps or calories expended. Due to the patient’s inability to obtain the necessary information to understand their conditions and detect illness, such as depression, objectively, methods for evaluating various mental disorders, such as the Montgomery–Asberg depression rating scale (MADRS) and observations, currently require a significant amount of effort on the part of specialists. In this study, a novel dataset was provided, comprising sensor data gathered from depressed patients. The dataset included 32 healthy controls and 23 unipolar and bipolar depressive patients with motor activity recordings. Along with the sensor data collected over several days of continuous measurement for each patient, some demographic information was also offered. The result of the experiment showed that less than 70 of the 100 epochs of the model’s training were completed. The Cohen Kappa score did not even pass 0.1 in the validation set, due to an imbalance in the class distribution, whereas in the second experiment, the majority of scores peaked in about 20 epochs, but because training continued during each epoch, it took much longer for the loss to decline before it fell below 0.1. In the second experiment, the model soon reached an accuracy of 0.991, which is as expected given the outcome of the UMAP dimensionality reduction. In the last experiment, UMAP and neural networks worked together to produce the best outcomes. They used a variety of machine learning classification algorithms, including the nearest neighbors, linear kernel SVM, Gaussian process, and random forest. This paper used the UMAP unsupervised machine learning dimensionality reduction without the neural network and showed a slightly lower score (QDA). By considering the ratings of the patient’s depressive symptoms that were completed by medical specialists, it is possible to better understand the relationship between depression and motor activity.

## 1. Introduction

As the second most common condition in the US, behind hypertension, depression is the fourth disease in the world to cause disability. Additionally, the COVID-19 epidemic has made things worse. Depressive symptoms rose from 8.5% to 27.8% throughout the quarantine period, according to [1,2]. Depression might become the most common disease as a result of this phenomenon, which would have a significant impact on healthcare costs and public health. Depression of the most serious severity is known as a major depressive disorder (MDD) [2]. Patients may exhibit symptoms including helplessness, depression, sadness, poor energy, sleep disturbance, low appetite, and interference with regular activities like work, study, or household chores for at least two weeks to receive an MDD diagnosis [3]. The prevalence of psychotic illnesses, on the other hand, is 3.89 per 1000, which is a comparatively low rate of disease [3,4,5].

Using on-body sensors to track one’s health has become prevalent in today’s society. People today gather a ton of information daily, which helps to significantly enhance their quality of life, monitor their fitness, or even quit bad habits. Because continuous records of heart rate and activity levels are routinely recorded, these data have great promise in addition to measuring daily steps or calories burned. The relationship between these activity statistics and other mental-health-related disorders, including mood or personality changes, an inability to handle stress or daily problems, and withdrawal from friends and hobbies, is becoming increasingly clear in the field of psychiatry [5,6]. Internal biological system disruptions are linked to mental health issues [6]. These are intricate systems, and it can be challenging to identify changes in them since the relationships between sensor data and mood are still poorly understood. Early warning signals, according to research, are frequently signaled by a phenomenon known as a critical slowing and take place during key transition times before rapid and visible changes in status [7]. Critical slowing down is characterized by a decreased capacity for self-restoration, i.e., a retarding of the system’s ability to recover from minor disruptions [8]. The pathologic state and the healthy state in depression and bipolar disorder may be interpreted as two distinct stable states that are abruptly separated from one another [9].

Certainly, the 24 h circadian clock is synchronized with many ultradian rhythmic cycles that last between two and six hours. This drives cyclical biological rhythms in connection with repetitive daily social rhythms [9,10,11]. Key symptoms of mood episodes [10] have been proposed as biological rhythmic rhythms that are out of sync [1]. Complex dynamical systems are time series of repeating biological rhythms and daily activities [11]. Complex dynamical systems are rarely categorized by straightforward linear models. Therefore, the traditional approach to analyzing and assessing motor activity recordings has been based on mathematical methods used in the study of non-linear, complex, and chaotic systems [12]. The capacity of ML to discover non-obvious patterns was utilized to largely correctly categorize mood states in a long-term heart rate variability study of bipolar individuals. In the study of data from complicated dynamical systems, ML approaches have shown promising outcomes [13]. Similar changes in cardiovascular autonomic functions have been discovered in manic individuals by nonlinear heart rate variability investigations [14,15,16]. Compared to heart rate data, accelerometer recordings are significantly noisier. However, time series of motor activity offer tremendous potential for many ML techniques. Techniques like neural networks and random forests [15] have shown encouraging potential for handling time series of activation data. However, results from high-quality analyses of critical variables should be taken seriously, at least when overfitting prevention strategies have been used [16]. The Random Forest algorithm’s ensemble learning technique is resistant to overfitting, and it can be seen as a woodland of decision trees, where different trees each focus on a different stochastic aspect of the data [17]. Decisions in decision trees are transparent, and lines of reasoning may be understood [18].

In connection with this, recordings of motor activity can be used to gauge the health of biological systems. Research demonstrates that depressive moods are associated with both decreased daytime motor activity and increased nighttime activity when compared to healthy controls [18,19]. Decreased motor activity and greater levels of activity variability have also been connected to bipolar depression [20]. In the realm of mental health, measurements of activity and mobility are currently a popular topic. In several investigations, sensors have been employed to monitor patients’ movements over time and relate them to self-reports or diagnoses [21]. The classic linear and nonlinear statistical methods are often used in these investigations to analyze the data. Among the stated findings [22], increasing skewness and autocorrelations, both indicators of a significant slowing down, are also reported [23]. As is clear, such data also have application potential for ML techniques, which are increasingly being applied in the area of psychology and psychiatry [23].

**Figure 1:**
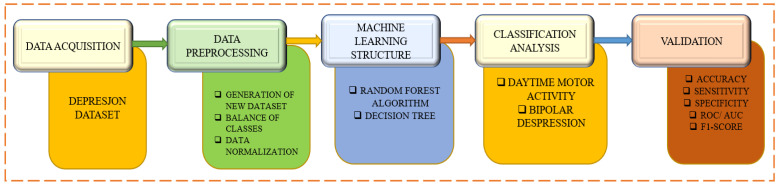
Bipolar depression detection and classification using machine learning and the depression dataset based on actigraphic registration of motor activity.

A depression dataset based on motor activity and machine learning is used in Figure 1 to identify and categorize bipolar depression. The first phase describes the data collection procedure from which the participants’ or patients’ motor activity records are gathered. Data pre-processing is involved in the second step, which is followed by the ML method with feature extraction and additional methods like random forest and decision tree. Beginning with daytime motor activity and bipolar depression, the categorization analysis method then starts. The various categorization models are assessed to complete the validation steps.

These databases face the two difficult problems listed below: Data in the medical profession are generally protected and difficult to obtain; data frequently comprise a small number of positive examples but considerably more bad ones, making it difficult to compare the results of different treatments (episodes are typically not the norm, and it is much easier to collect normal data compared to relevant cases). By making it completely available for research and having a disproportionately high proportion of depression cases compared to control patients, the dataset described here, called Depresjon after the Norwegian word for depression, tries to solve these two issues. Therefore, the main contributions of this study are as follows:

- The study provides a unique and open dataset consisting of sensor data collected from depressed patients as well as healthy individuals. This dataset offers valuable resources for further research in the field of depression and related disorders.
- The dataset includes a significant number of patients with both unipolar and bipolar depression, allowing for a comprehensive analysis of different forms of depression. The presence of healthy controls further enhances the comparative aspect of the study.
- The study employs machine learning (ML) algorithms to differentiate between “bad days” and “good days” based on motor activity data. This analysis provides insights into the relationship between motor activity patterns and depressive symptoms.
- The study suggests evaluation measures that can be utilized in future research on depression detection and classification. These measures serve as a reference point for assessing the effectiveness of different methods and algorithms.

This paper is organized as follows: In Section 2, the literature review is presented in great detail. Section 3 provides a dataset, while Section 4 outlines the methodology. The results are covered in Section 5 and the discussion is presented in Section 6. The assignment is ultimately concluded in Section 7.

## 2. Literature Review

In terms of size reduction and energy efficiency, sensor technology has advanced significantly over the past few years. These technological advancements have sped up the creation of new categories of technology, like smartphones and smartwatches with potent sensing capabilities. Lately, experts have begun investigating fresh strategies for employing those gadgets to covertly and continuously observe consumers. The long-term collection of objective data has also been made possible by these technologies. Understanding users’ surroundings and actions through data analysis utilizing machine learning techniques paves the way for the development of useful systems like activity identification, indoor positioning, and fitness monitoring. The field of mental healthcare has a lot of potential for these pervasive technologies. Early research has focused on the use of sensing devices to automatically track patient sadness. For instance, Ref. [3] uses a wrist-worn activity monitoring unit to track persons with late-life depression and discovers that their physical activity was lower than that of healthy controls. In a different study, Ref. [5] uses smartphone sensors to track bipolar illness patients and discover that the more severe the depression symptoms, the fewer outgoing calls they registered and the less they responded to incoming calls. Based on cell tower IDs, they also discover that sad patients moved less. In contrast to the previous two studies, our work uses machine learning to automatically distinguish between depressed and nondepressed people using statistical variables generated from sensor data. A depressed mood is associated with both lower daytime motor activity and increased night-time motor activity, as shown by systematic reviews of the use of actigraphy in research on depression [5]. Bipolar depression shares traits with other types of depression, such as lower motor activity and higher levels of activity variation [6]. Machine learning has also been utilized in several types of research to recognize depressive conditions. For instance, Ref. [7] classifies manic and depressed states in bipolar patients using smartphone data such as acceleration, sound, and position, with a 76% accuracy rate using a Naive Bayes classifier. Our strategy is distinct since it focuses more on the diagnosis and seeks to establish whether a patient is depressed or not. Additionally, it has been shown that social media can be utilized to identify sadness, for example, by analyzing submitted Instagram photos [8].

Another intriguing approach is that of [8], who describe how psychological treatments using a smartphone as a clinical tool could reduce anxiety in schizophrenia patients. Tommy et al.’s analysis provides information on psychiatric patients’ interest in and use of mobile applications to monitor their mental health problems. To manage their disease, 50% of patients across all age groups plan to utilize mobile applications to track their mental health, according to the survey’s findings. Activity detection at different levels of activity abstraction is described in Stockings et al.’s systematic review of numerous works [13] that focus on the use of mobile phone sensors to detect human behavior characteristics, and characterizes health-related activities, such as physical activity and sleep. In addition to being used in applications, these devices also have several embedded sensors that have been applied in a variety of fields [14,15], such as activity recognition [16] and, in particular, an activity that helps detect mental health issues [17]. To demonstrate how inertial sensors and GPS traces can be used as measurement instruments in mental diagnosis, Ref. [15] uses a methodology based on feature extraction of physical motion levels and trip patterns and a classification analysis using a naive Bayes algorithm. Using submitted Instagram photographs and the random forest method, Ref. [18] determines participants who are depressed. A study on smartphone data [19] proposes the classification of manic and depressive episodes in bipolar patients. Bipolar patients are categorized by [20] using audio, motor activity, and questionnaires. Using data on motor activity, Ref. [21] suggests a method for identifying patterns in schizophrenia and depression.

Ref. [22] discusses research on the utilization of combining biomarkers from other techniques, such as motor activity based on actigraphy data, suggesting that patient distinction based on these biomarkers improves the diagnosis of depressed individuals. Actigraphy activity levels are used by [23,24,25] to analyze the psychomotor changes that take place during depressive episodes to assess how well the treatment for depression is working. They conclude that early alterations in the basic activity and psychomotor speed can be used to assess the efficacy of the therapy in depressed patients. Ref. [26] finds that the actigraphy data contain information that enables evaluation of a subject’s depression condition in their research on actigraphy data using machine learning to categorize depressed persons. Ref. [27] proposes a review to help people with depression select self-help apps. Given the weak level of commitment to the core elements of the CBT and BA models, the value of applications that offer cognitive behavioral therapy (CBT) or behavioral activation (BA) is questioned. It was possible to conclude that using higher levels of scientific, technological, and legal knowledge is necessary to increase the credibility of the apps for people with depression [28].

**Figure 2:**
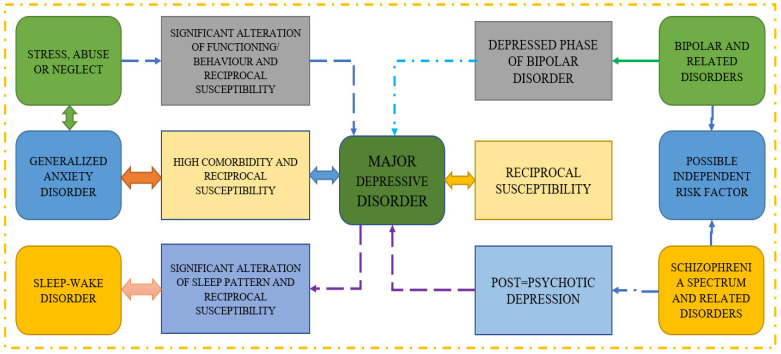
Approaches used in several publications on Major Depressive Disorder.

On the other hand, a layered and hierarchical model for the transformation of raw sensor data into indicators of behaviors and states related to mental health is provided by Falciani et al. in their assessment of sensing research on mental health [29]. Reference [30] provides a review of the study that uses screening questionnaires, public sharing on Twitter, and participation in an online forum to predict mental illness. They conclude that passive activity on social media can be monitored to identify sad or at-risk persons using automated detection approaches. Moreover, the methodologies employed in several reference papers on major depressive disorder are shown in Figure 2, which also demonstrates how various disorders can lead to MDD. It is well established that reciprocal susceptibility and generalized anxiety disorders are two-way illnesses that can lead to serious problems.

Nie et al. [31] focus on long dialogue emotion identification in this study and present a system that makes use of commonsense knowledge graph guidance. In lengthy dialogues, they address the problem of understanding emotions and demonstrate the effectiveness of their technique. Their method improves the accuracy and comprehensiveness of emotion identification in dialogue-based multimedia content by using commonsense knowledge. Wang et al. [32] conduct a randomized controlled trial to investigate the efficacy of transcranial alternating current stimulation (tACS) in the treatment of depression. They investigate the possibility of tACS as a non-invasive neuromodulation technology and provide useful information about its therapeutic benefits. The findings add to the growing amount of research on innovative methods to depression management.

Liu et al. [33] present an overview of current breakthroughs in pulse-coupled neural networks (PCNNs) and their image processing applications. They address the underlying ideas and architectures of PCNNs, as well as their use in a variety of image-related tasks. The review is an excellent resource for scholars and practitioners interested in using PCNNs for image analysis and processing. Liu et al. [34] concentrate on region-aware picture captioning and present a novel approach based on interaction learning. They solve the difficulty of producing accurate and informative captions by incorporating image-level interactions. Their technology improves performance in generating region-aware captions and adds to the advancement of picture captioning. Xie et al. [35] provide a study on the prediction of unexpected rainy scenarios and propose an integrated strategy that integrates decision makers’ emotions, dynamic Bayesian networks, and DS evidence theory. They underline the need for addressing emotional variables in natural disaster decision-making processes. Their research contributes to the development of more effective techniques for dealing with unexpected rainstorms. The summary of the state of the art techniques are listed in the below Table 1.

The review of the literature identifies several unresolved issues in sensor-based mental health monitoring [37,38]. First off, there are not many thorough, standardized methodologies for combining sensor data with machine learning. The development of standardized methods that can be widely used and compared between studies should be the main goal of future research. Second, there is not much attention paid to diagnostic models that use sensor data. To increase the precision and diagnostic capabilities of these models, more research is required. Thirdly, the range of behavioral and physiological indicators collected for monitoring mental health can be expanded by integrating multiple sensor modalities. To increase accuracy and reliability, research should investigate the pairing of various sensors. Additionally, there is a need to broaden the application of sensor technology to a wider range of mental health conditions, as the majority of current studies primarily concentrate on particular mental health conditions like depression and bipolar disorder. Since the use of ubiquitous sensing technologies raises questions about responsible data collection and usage, ethical and privacy considerations also need to be taken into account. Finally, the viability of sensor-based mental health monitoring systems depends on their validation in real-world settings and scalability. To assess the efficacy and scalability of these technologies in practical settings, extensive trials and feasibility studies are required. The development of novel interventions and the advancement of sensor-based mental health monitoring can both benefit from filling in these research gaps.

This study aims to fill the research gaps found in the literature review by creating a model for depression detection and classification using sensor data. The research offers a unique dataset made up of sensor data gathered from depressed patients, including both unipolar and bipolar depressive patients, as well as healthy controls. Additionally, the dataset contains demographic data. The relationship between motor activity and depression can be examined in greater detail thanks to this extensive dataset. A model is trained in the study’s experiment using machine learning methods and UMAP dimensionality reduction. In the second experiment, the model achieves an accuracy of 0.991, demonstrating its efficacy in differentiating between people with and without depression. To obtain the best results in depression detection and classification, UMAP and neural networks are combined. The proposed model improves our understanding of the relationship between depression and motor activity by taking into account the ratings of depressive symptoms completed by medical professionals. Compared to conventional techniques that rely on judgments and observations, it offers a method for evaluating and detecting depression that is more objective and effective. Detailed information of the proposed model is discussed in the subsequent sections.

## 3. Dataset

The actigraphic registration of motor activity revealed a more ordered behavioral pattern in schizophrenia than in major depression, with the study of [39] serving as the initial source for the data. It comprised the actigraph recordings of motor activity from 32 healthy individuals and 23 individuals with schizophrenia (unipolar and bipolar) who were treated as inpatients or outpatients, respectively. These individuals are referred to in the study as conditions and controls. Actigraphy is the study of human sleep and activity patterns. The participants’ right wrists were adorned with an actiwatch, which the researchers utilized to record that information. In contrast to the recordings of the healthy participants, the patient’s data were also attached, along with personal details about the patient, such as age, gender, education, employment, marital status, the presence or absence of melancholy, the type of affiliation (bipolar I, bipolar II, unipolar depressive), and the assessment of their depressive symptoms using the Montgomery–Asberg Depression Rating Scale (MADRS) [40].

The dataset used for the implementation was derived from a study on the actigraphic registration of motor activity in individuals with schizophrenia and major depression. The dataset comprised actigraph recordings of motor activity from 32 healthy individuals (referred to as controls) and 23 individuals with schizophrenia (both unipolar and bipolar). The individuals with schizophrenia were treated either as inpatients or outpatients.

### 3.1. Dataset Attributes

Many characteristics in the dataset contained important details about the participants and their motor activity patterns. These qualities consisted of the following

- Actigraph recordings: Actiwatches worn on the participants’ right wrists were used to record their motor activity, and these recordings are referred to as “actigraphs”. Actiwatches are tools that track and record motion and provide unbiased assessments of motor activity. These recordings provide information about the individuals’ sleep habits, rest states, and general levels of activity over the course of the experiment.
- Personal information: The dataset included participant personal information such as age, gender, education, employment, marital status, and kind of affiliation. Age helps participants identify their life stage, whilst gender can help investigate potential differences in motor activity patterns between males and females. Education, occupation, and marital status provide information on participants’ socioeconomic status and daily routines, which may influence their motor activity.
- Melancholy: This feature reflects whether or not the individuals experienced melancholy, which is characterized by sadness, low mood, or depression. It gives information on the participants’ mental health and allows for the investigation of potential correlations between melancholy and motor activity patterns.
- MADRS (Montgomery–Asberg Depression Rating Scale): The MADRS scale is a popular instrument for evaluating depressive symptoms. It provides a standardized measure of the severity of depression, taking into account aspects such as sorrow, pessimism, sleep disorders, and difficulty concentrating. The inclusion of MADRS assessments in the dataset enables researchers to look into the link between depressive symptoms and motor activity patterns.

These factors, taken together, provide a full picture of the participants’ features, mental health state, and motor activity patterns. Researchers can acquire insights into the association between motor activity and mental health problems such as depression and schizophrenia by evaluating and exploring these aspects. Furthermore, these characteristics allow for additional research into potential factors impacting motor activity, such as age, gender, and socioeconomic considerations.

### 3.2. Data Visualization

**Figure 3:**
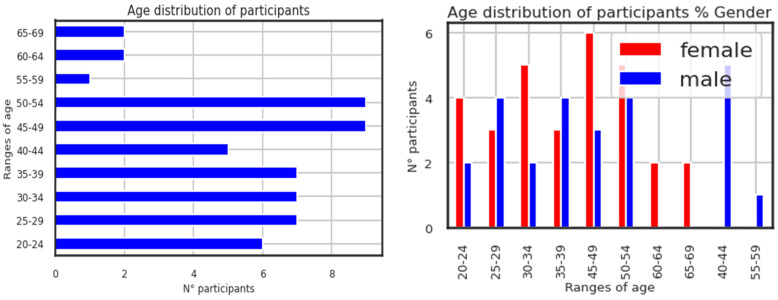
Age distribution of the participants.

The right-hand figure on the page illustrates the age distribution of participants by the proportion of genders. It is shown below in Figure 3, which displays the participants’ ages. Although the selection procedure was not skewed to utilize the age feature as the data processing, it is useful in data exploration. The participants (conditions and controls) range in age from 20 to 69 years old, with 30 females and 25 males. Higher N° participants are found in the participant age groups of 45 to 54.

**Figure 4:**
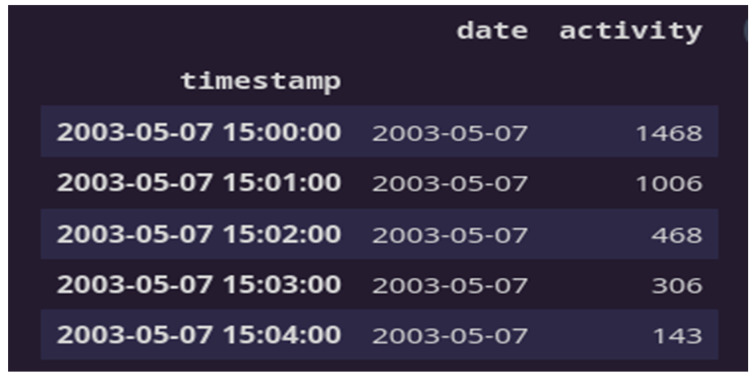
Data sample from actigraphy recordings of a participant.

The data sample gathered from the participants’ actigraphy recordings is displayed below in Figure 4. Additionally, during the experiment, data were gathered every minute at a frequency of 32 Hz and for movements greater than 0.05 g. This figure shows the activities of four subjects throughout the course of 24 h; one is healthy, and the other three exhibit the three forms of depression recognized in this study. It can be seen from the recordings alone how a healthy participant’s activity and rest states can be separated from those of the sad patients.

**Figure 5:**
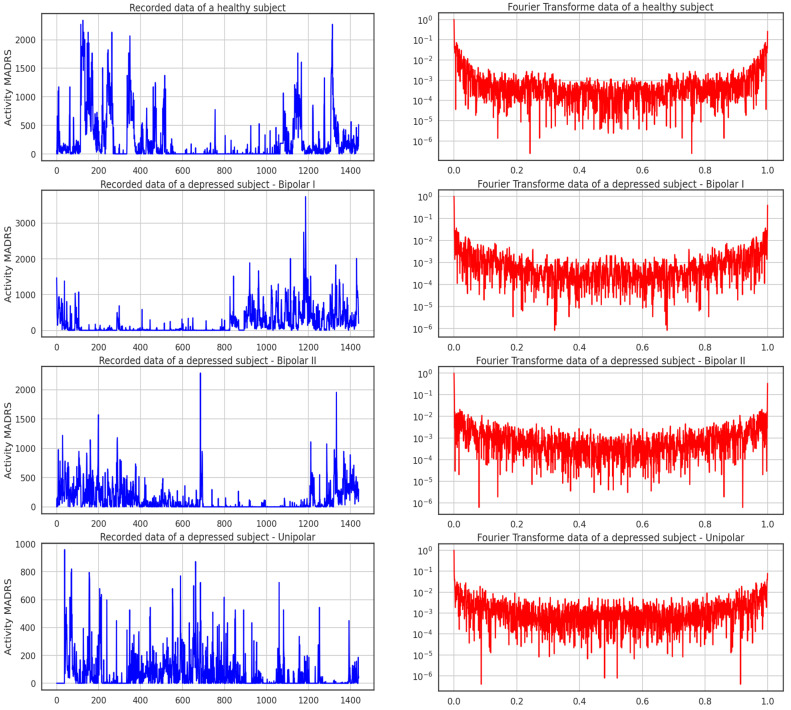
Activity recorded over 24 h of control and condition participants.

Figure 5 shows the activity logged over 24 h by participants in the control and condition groups, where blue and red activities are related to the recorded data of a healthy subject and the Fourier transform of a healthy subject, respectively. Here, data for bipolar I, bipolar II, and unipolar are all compiled.

**Figure 6:**
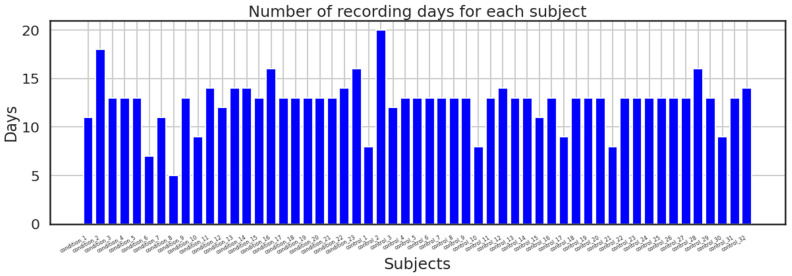
Number of recording days.

In addition, the dataset made available to the public was split into two directories: one folder contains recordings from the control group along with date, timestamps, and activity measures for each participant in a CSV file; the second directory contains recordings from the condition group with the same parameters as the control group; and lastly, the dataset also includes a score. The above-mentioned participants’ data are contained in the CSV file. The majority of the recordings were recorded over a period of 13 days; however, others took longer or shorter. Figure 6 shows how many days were spent recording for each subject.

## 4. Methodology

The main objective is to determine whether or not a person is depressed. The following method is recommended to achieve this goal: Let us denote the participant who gathered the most data as “i”, and “di” as the total number of days during which each participant collected data. From each day, statistical data were extracted to create feature vectors for each participant. To prevent overfitting, a Leave-One-Out validation technique was employed. This technique involved training the classifier using all the data from all other users (excluding “i”) and testing it using the data from “i”. A vector of predictions was initially collected from the trained classifier to determine the final classification for a specific person—whether they are depressed or not. Each entry relates to the forecast for a specific day.

**Figure 7:**
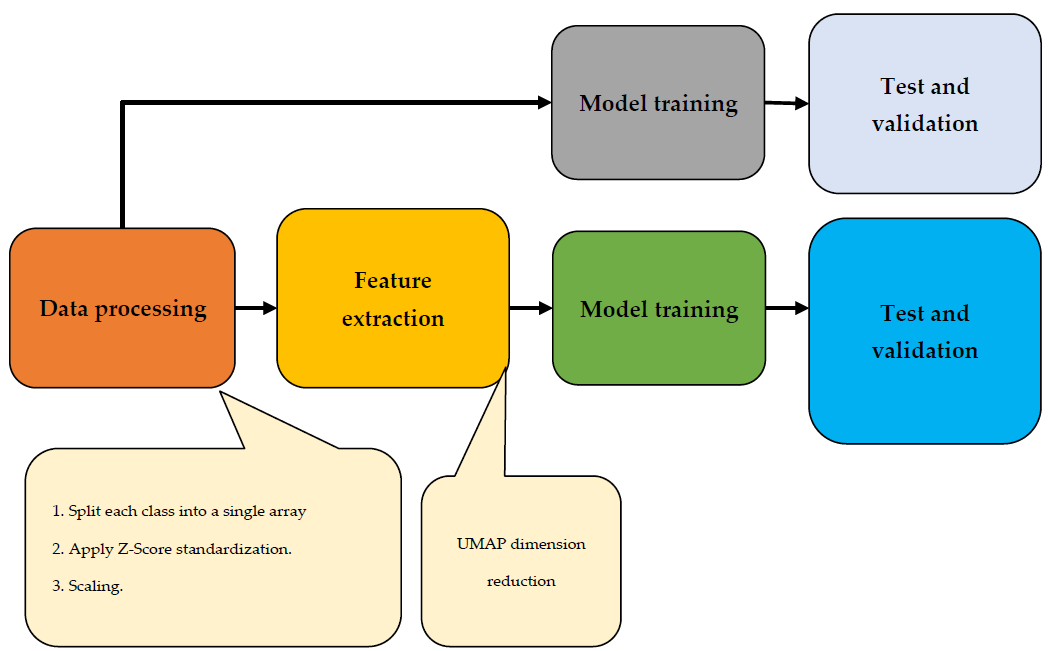
Workflow chart of the implementation of the solution.

The majority vote, which outputs the most frequent forecast from the group, determines the final label. This study utilized various class-balancing approaches due to the unbalanced nature of the data. Specifically, two oversampling methods were applied to enhance the minority class data. The first method involved duplicating randomly selected data points through random oversampling. The second method, called SMOTE, generated synthetic samples by selecting nearby points that were similar to each other. Two different ML classifiers, an RF and a DNN, were tested alongside a baseline classifier that assigned random class labels based on previous probabilities. The solution’s implementation is depicted in the workflow diagram shown in Figure 7. Following the initial data processing step, the feature extraction process took place. Subsequently, the data underwent testing, training, and validation phases. To ensure accurate results and facilitate comparison, two trials for this image were planned: one with UMAP dimension reduction and one without.

### 4.1. Data Preprocessing

The data preprocessing phase plays a crucial role in preparing the dataset for further analysis and modeling. In this study, a series of preprocessing steps were applied to ensure the data’s quality, consistency, and compatibility with the proposed system. The data preprocessing pipeline consisted of the following steps:

#### 4.1.1. Leave-One-Out Validation Technique

The proposed system implemented two strategies to address the issues of overfitting and a small dataset. Firstly, a Leave-One-Out validation technique was employed to prevent overfitting. This technique involved training the classifier using data from all participants except the one being tested. By evaluating the classifier’s performance on unseen data and avoiding bias toward specific participants, overfitting was mitigated.

#### 4.1.2. SMOTE

The system tackled the problem of class imbalance in the dataset. Some classes had significantly fewer samples, which could lead to biased predictions. To address this, two oversampling methods were utilized: random oversampling and the Synthetic Minority Over-Sampling Technique (SMOTE). Random oversampling duplicated randomly selected data points from the minority class, while SMOTE generated synthetic samples by interpolating between similar data points. These techniques balanced the class distribution and provided the classifier with a more representative training dataset.

In addition to the aforementioned steps, several other data preprocessing techniques were applied to ensure the quality and compatibility of the dataset. Data cleaning played a vital role in the initial phase, involving a thorough examination of the raw dataset to identify and handle missing or erroneous values. Various imputation techniques, including mean imputation, median imputation, or regression-based imputation, were employed to fill in the missing values based on the nature and distribution of the data. Statistical methods were utilized to detect outliers, and appropriate strategies such as removing outliers or replacing them with suitable values were implemented. Moreover, categorical variables were transformed into numerical representations to facilitate their integration into the modeling process.

### 4.2. Feature Extraction

The data were gathered and divided into 4 arrays for each of the model’s 4 classes—healthy, bipolar I, bipolar II, and unipolar—because each class was considered. The data processing left the time series in the shape of an array of activity values, truncated the arrays into the same size ts and smaller arrays, then concatenated them into one array of shape [N, ts] with N samples, each having a ts data sample. As was previously mentioned, the dataset is a set of time series of controls and conditions, all recorded with a timestamp of 1 min. This created a data sample of ts dimension. The classes of the dataset are listed in the below Table 2. 

Once that finished, skewed data were transformed using the SciPy library using a z-score transformation to roughly adhere to normality:

(1) $$X=\frac{\left(x-µ\right)}{\sigma }$$

The z-score can be calculated using Equation (1) above for a given number from any distribution; it is always calculated by taking *X*, minus the distribution’s mean, and then dividing by the distribution’s standard deviation [41]. Each sample was labeled according to the tape it was derived from in order to produce a rigorous target value, resulting in 4 classes overall. The authors of [42] applied an unsupervised machine learning dimensionality reduction (UMAP) to improve performance, in contrast to the work of [43,44] and others, but only two classes—healthy and depressed—were considered; no classification of the depressed type was used. Four courses were considered in this essay, as was previously mentioned.

The UMAP technique is a dimensionality reduction algorithm that preserves the structure and relationships of high-dimensional data when projecting them to a lower-dimensional space. It involves several key steps: nearest-neighbor search to construct a graph, fuzzy-simplicial set approximation to approximate the topological structure, optimization and embedding to obtain a low-dimensional representation by minimizing the discrepancy between distances, and continuation and hierarchical structure for exploring different scales and identifying hierarchical relationships. The UMAP architecture combines these steps to generate a compact representation of the input data for visualization, clustering, or other data analysis tasks.

**Figure 8:**
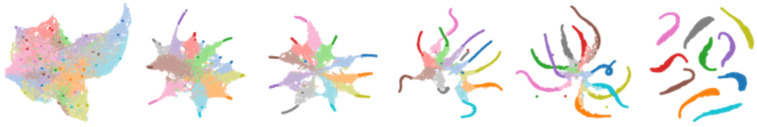
Illustration of multidimensional data transformed using the UMAP technique.

The Uniform Manifold Approximation and Projection (UMAP) technique is described in Figure 8, which shows multidimensional data transformed using the UMAP technique. UMAP assumes that the available data samples are evenly (uniformly) distributed across a topological space (manifold), which can be approximated from these finite data samples and mapped (projected) to a lower-dimensional space.

Finding a low-dimensional representation and learning the manifold structure make up the majority of the UMAP approach.

- Finding the nearest neighbors is the first stage in learning the structure of a manifold. Next, it moves on to linking those nearest neighbors to create a graph, which will produce an approximation of a manifold. This process takes place in high-dimensional space.
- The process of locating a low-dimensional representation entails projecting the approximate manifold onto a lower-dimensional space.

**Figure 9:**
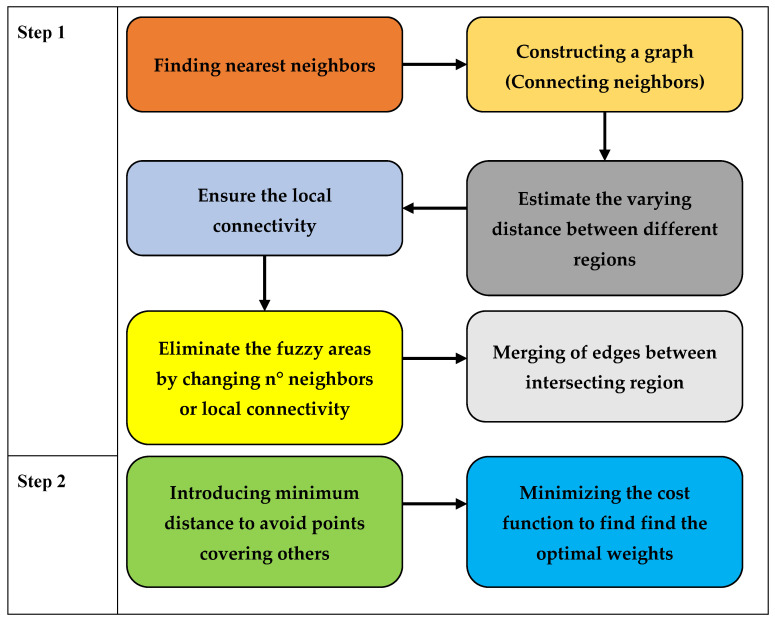
Schematization of the UMAP technique.

Figure 9 illustrates the UMAP approach schematized. Above it, the first step involves finding the closest neighbors by building a graph, estimating the varying distance between various regions while maintaining local connectivity, removing the fuzzy areas by changing the n-degree neighbors, and merging the edges between intersecting regions to complete the step. Additionally, the UMAP approach does away with the minimum distance in step 2 to prevent points from converging. To determine the ideal weights, it finally minimized the cost function [45].

To effectively apply the UMAP (Uniform Manifold Approximation and Projection) algorithm in Python, the UMAP-learn library was utilized. This library provides pre-trained pipelines that are compatible with NumPy data and Pandas [36]. The following steps outline the implementation process:

- Import data: The first step is to import the data that will be used for UMAP. This can be performed by loading the data from a file or retrieving it from a database, depending on the data source.
- Set desired parameters: UMAP offers various parameters that can be set according to the desired outcome. For example, you can specify the maximum number of items to be included in the final result or choose the algorithm used to transform the data. These parameters can be adjusted to customize the UMAP process to fit the specific requirements of the analysis.
- Train UMAP: Once the desired parameters are set, the UMAP algorithm needs to be trained on the original data. This involves feeding the data into the UMAP model and allowing it to learn the underlying patterns and structure in the data. The training process aims to create a low-dimensional representation of the data that preserves the relevant information.
- Apply UMAP on the data: After the UMAP model has been trained, it can be applied to the original data. This step involves transforming the data into a lower-dimensional space, where each data point is represented by a set of coordinates. UMAP leverages manifold learning techniques to map high-dimensional data onto a lower-dimensional space while preserving the inherent structure and relationships among the data points.
- Data visualization and analysis: The final transformed data obtained from UMAP can be utilized for data visualization and analysis. The lower-dimensional representation allows for easier visualization, as the data points can be plotted in a reduced space. This visualization helps in understanding the structure, patterns, and clusters present in the data. Additionally, the transformed data can be further analyzed using various techniques and algorithms tailored for lower-dimensional spaces.

**Figure 10:**
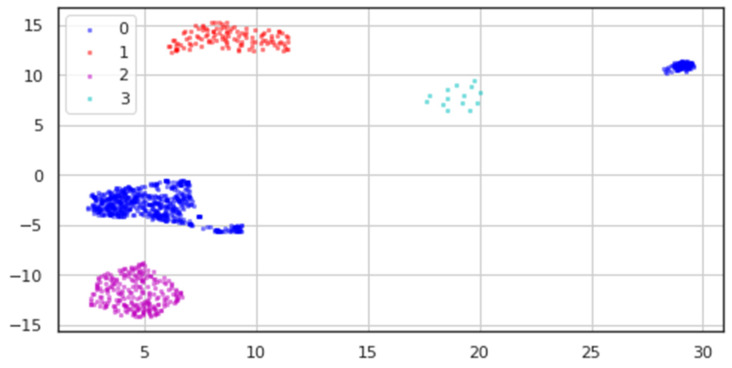
Resulting UMAP dimensionality reduction of the dataset.

All classes’ processed data underwent dimensionality reduction, resulting in the two-dimensional data depicted in Figure 10, where blue dots stand for the Healthy class, red points for Bipolar I, magenta points for Unipolar, and cyan points for Bipolar II.

We can see from the modified data in Table 3 how sparse the data for the Bipolar II (labeled 3) class is, in contrast to how distinct the other classes are.

### 4.3. Model Training

After applying dimensionality reduction, the data were randomly divided into three sets for training, testing, and validation. The training set comprised 70% of the data, the testing set contained 20%, and the remaining 10% was allocated for validation. This particular ratio is commonly employed by researchers to assess the performance of the proposed system. Cross-entropy was utilized as a loss function with the Adam optimizer for the classification model’s neural network, which included 52 parameters. Accuracy score, F1-score, and Cohen Kappa were used as metrics to assess the model’s performance.

- Cross-entropy loss, also known as log loss, assesses how well a classification model performs when producing a probability between 0 and 1.
- Accuracy score: This score assesses the model’s performance by comparing the ratio of true positive to true negative outcomes among all created predictions.
- F1 score: comparable to accuracy score, but requires fewer observations.
- Cohen-Kappa Score: Calculations that account for chance—the degree of agreement between qualitative assessments made of identical things by two observers or methods.

**Figure 11:**
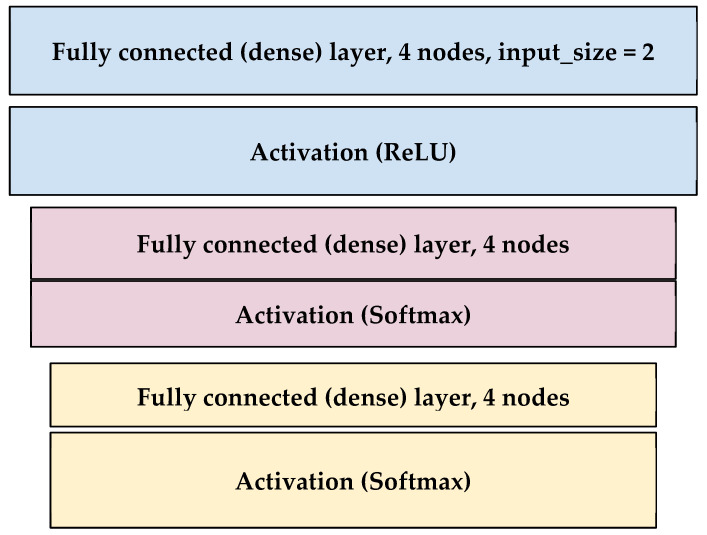
Model architecture summary.

Figure 11 model is an example of a trained model with more than 100 epochs and a batch size of 30 samples. According to the type of data used, experiment 1’s input size in layer 1 was 1440 but experiment 2’s input size was 2 (reduced or raw).

The proposed system’s hyperparameter adjustment was critical in optimizing its performance. To explore the hyperparameter space and discover the best combination of values for the model, a methodical methodology was used. Grid search, random search, and Bayesian optimization techniques were used to find the best hyperparameters. The tuned hyperparameters differed based on the model utilized in the proposed system. Parameters such as the number of trees, the maximum depth of each tree, and the number of features examined for each split, for example, were modified in a Random Forest classifier. The learning rate, batch size, number of hidden layers, and number of neurons in each layer were all changed in a DNN.

The tuning procedure entailed training and assessing the model numerous times with various hyperparameter combinations, comparing their performance, and selecting the set of hyperparameters that produced the best results. The goal of this iterative procedure was to discover the hyperparameter configuration that maximized the model’s performance and generalizability. The suggested system was able to fine-tune its models, maximize their performance, and obtain superior outcomes when compared to using default hyperparameter values by performing hyperparameter tweaking.

## 5. Results

In this study, two experiments were conducted to evaluate the performance of the model. In Experiment 1, various metrics including accuracy, F1-score, and Cohen Kappa were used to assess the model’s performance. These metrics provide insights into the model’s accuracy, precision, recall, and agreement with the actual labels. In Experiment 2, the same metrics were employed to evaluate the model’s performance. The focus was on comparing the results with other studies, shedding light on the strengths and weaknesses of the model concerning existing approaches. The analysis of the confusion matrix revealed interesting findings, particularly regarding class imbalances and misclassifications, with a notable impact on the Bipolar II class due to the limited number of samples available. This highlights the challenges of training a model when there is an unequal distribution of classes and the importance of addressing such imbalances in future studies. Furthermore, additional statistical analysis was conducted to gain a deeper understanding of the data. Various statistical measures such as mean, number of zeros, skewness, and standard deviation were computed. These measures provided insights into the characteristics of the patients and their behaviors, suggesting a higher level of activity and a significant decrease in MADRS scores over time.

### 5.1. Experiment 1

**Figure 12:**
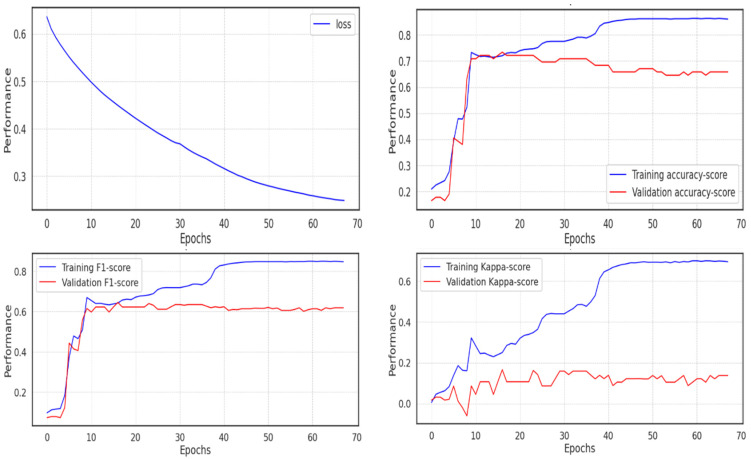
Model performance on the validation data in experiment 1.

Using the unprocessed data, the first experiment was run (not the extracted features with UMAP). Due to the implementation of the Adam optimizer, which ends training once the network approaches the local minimum and leaves no room for improvement, fewer than 70 of the 100 epochs of the model’s training were completed. The cross-entropy loss decreased and almost reached 0.2, but the validation score performed poorly in comparison to the training score, passing 0.8 in accuracy and F1 score, whereas the validation score remained around 0.6, and the Cohen Kappa score did not even pass 0.1 in the validation set, due to the imbalance in the class distribution, as shown in Figure 12.

The metrics and validation score for the model are shown in Table 4 below. If the f1-score is 0.56, the accuracy is roughly 0.63, and the Cohen Kappa validation score is 0.105.

### 5.2. Experiment 2

**Figure 13:**
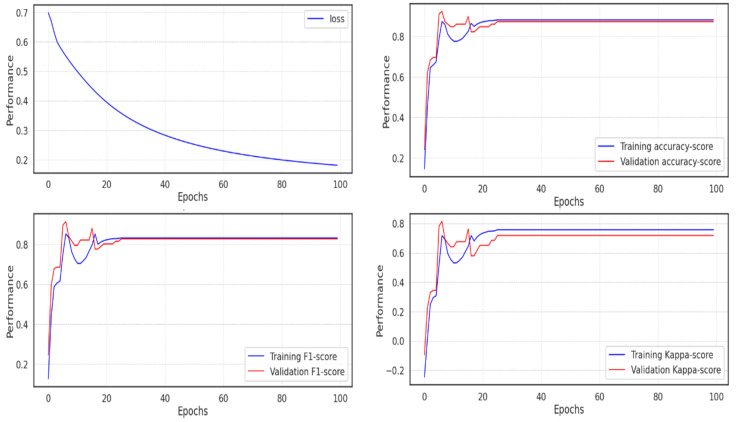
Model performance on the validation data in experiment 2.

In the second experiment, the model soon reached an accuracy of 0.991, which can be seen as expected given the outcomes of the UMAP dimensionality reduction, where it is simple to determine the categorization of the various classes. The majority of scores peaked in about 20 epochs, but because training continued during each epoch, it took much longer for the loss to decline before it fell below 0.2, as seen in Figure 13.

The metrics and validation score for the model in experiment 2 are displayed in Table 5 below, where the Cohen Kappa validation score is 0.977, the f1-score is 0.99, and the accuracy is around 0.991.

### 5.3. UMAP, Neural Network

The findings of the two trials were as expected: UMAP and neural networks worked together to produce the best outcomes. While Garcia-Ceja et al. [43] used a variety of machine learning classification algorithms, including the nearest neighbors, linear kernel SVM, radial basis function kernel (RBF) SVM, Gaussian process, decision tree, and random forest, Price et al.’s paper [41] used the UMAP unsupervised machine learning dimensionality reduction without the neural network in forward and showed a slightly lower score (QDA). None of the algorithms had a score greater than 0.727. In Zanella-Calzada et al.’s research [4], statistical feature extraction was performed to feed a random forest classifier, which produced a score of 0.919 in Table 6.

**Figure 14:**
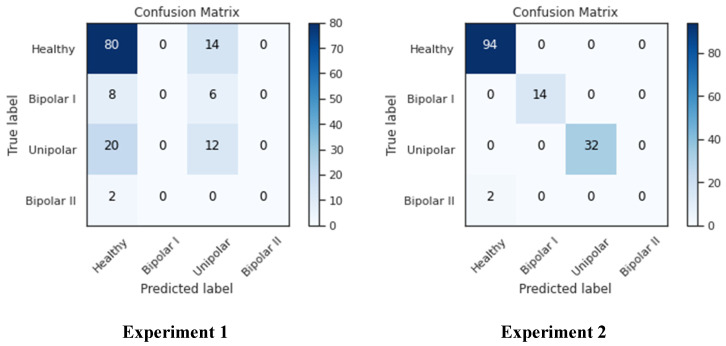
Confusion matrixes for both experiments.

As seen in Figure 14, the confusion matrix for both studies showed that the healthy-labeled samples had the maximum presence, while the bipolar II samples had essentially no presence and the remaining classes were in the middle. This brings us back to the nature of the data source; as was previously demonstrated, there were only 14 samples in the bipolar II class, and only two samples were left in the testing set after the data had been divided among the training, testing, and validation sets. This has an impact on how well the network trains because with fewer samples, it is more difficult to identify the data, as seen in the left-bottom cell, where Bipolar II samples were mistaken for healthy samples. As seen in columns one and three of the confusion matrix, many samples in experiment 2 were incorrectly classified as “unipolar” or “healthy”, whereas in experiment 2, all classes except Bipolar II were correctly classified. This can be attributed to the size of both classes, which are larger than the other two classes (Bipolar I and Bipolar II).

Numerous statistics were computed, such as the mean, number of zeros, skewness, and standard deviation, to enhance our understanding. These figures allowed us to see that the patients seemed to be significantly more active than the average of the patients with the other conditions. The fact that this was more extreme than it was for the other patients was more evidence of this. Taking a look at the patients’ MADRS scores also showed that from the time of admission to the time of release, these patients’ MADRS scores generally decreased significantly. This can be due to improved treatments or medications that were not included in the dataset. Since it appears that the five patients who were routinely misclassified by the MADRS scale share a trait but not with the controls or the patients with the other conditions, it might be interesting to classify patients in more than just depressed and non-depressed classes for future studies. To gain a better grasp of the classification performance, the MADRS scores for the controls should also be gathered for future datasets.

## 6. Discussion

ML has been used to develop an efficient way to classify more than one mental disorder against healthy controls using motor activity. The main idea of this work was to assemble an efficient model to classify multiple types of mental illness, rather than performing a binary classification between disease and healthy controls. Approaches were also presented to classify patients as depressed or non-depressed based on the motor activity data collected through actigraphy devices. The actigraphic registration of motor activity revealed a more ordered behavioral pattern in schizophrenia than in major depression. Berle et al. [39] studied 32 healthy individuals and 23 individuals with schizophrenia (unipolar and bipolar) that were treated as inpatients or outpatients. In contrast to the recordings of the healthy participants, the patient’s data were also attached, along with personal details about the patient, such as age, gender, education, employment, marital status, the presence or absence of melancholy, the type of affiliation (bipolar I, bipolar II, unipolar depressive), and the assessment of their depressive symptoms using the Montgomery–Asberg Depression Rating Scale. Moreover, fewer than 70 of the 100 epochs of the model’s training were completed. Using the unprocessed data, the first experiment was run. The Adam optimizer, which ends training once the network approaches the local minimum, left no room for improvement. The cross-entropy loss decreased and almost reached 0.2, but the validation score performed poorly in comparison to the training score. Table 4 displays the model’s metrics and validation score. The accuracy was approximately 0.63 and the Cohen Kappa validation score was 0.105 if the f1-score was 0.56. In the second experiment, the model quickly attained an accuracy of 0.991, which is to be expected given the results of the UMAP dimensionality reduction, where it is easy to identify the various classes’ classifications. The majority of scores peaked at 20 epochs on average, but because training persisted during each epoch, it took much longer for the loss to decrease before it dropped below 0.2. Table 5 shows the metrics and validation score for the model used in Experiment 2, where the accuracy was roughly 0.991, the f1-score was 0.99, and the Cohen Kappa validation score was 0.977. 

The two experiments’ results confirmed what was predicted: the best results were obtained when UMAP and neural networks collaborated. Price et al.’s paper [41] used the UMAP unsupervised machine learning dimensionality reduction without the neural network in forward and showed a marginally lower score compared to Garcia-Ceja et al. [43] who used of a variety of machine learning classification algorithms, including the nearest neighbor, linear kernel SVM, radial basis function kernel, SVM, Gaussian process, decision tree, and random forest (QDA). A score of more than 0.72 was not achieved by any algorithm. Moreover, the confusion matrix for both studies showed that the healthy-labeled samples had the maximum presence, while the bipolar II samples had essentially no presence. This can be attributed to the size of both classes, which are larger than the other two classes. Many samples in experiment 2 were incorrectly classified as “unipolar” or “healthy”. The patients’ mean, number of zeros, skewness, and standard deviation (std) were calculated with the aid of these statistics. This can be due to improved treatments or medications that were not included in the dataset. Taking a look at the patients’ MADRS scores also showed that from the time of admission to the time of release, these patients’ MADRS scores generally decreased significantly. With the aid of these statistics, it can be seen that the patients appeared to be much more active than the average of the other condition patients.

The proposed system underwent various optimizations for better performance. Leave-One-Out validation prevented overfitting, and the class imbalance was addressed using random oversampling and SMOTE. Multiple classifiers (RF and DNN) were tested, and UMAP was used for dimensionality reduction. Model training utilized training, testing, and validation sets, with evaluation metrics such as accuracy, F1-score, and Cohen Kappa. Results were compared with other studies, showcasing the system’s performance and contributions.

The study has several limitations that should be acknowledged. Firstly, the sample size was relatively small, consisting of 32 healthy controls and 23 depressed patients, which may limit the generalizability of the findings. Secondly, the class distribution within the dataset was imbalanced, particularly in the Bipolar II class, which can affect the model’s performance and lead to biased results. Additionally, the model’s training in Experiment 1 was incomplete, potentially impacting its performance. Another limitation is the lack of comparison with established gold-standard diagnostic methods for depression, limiting the evaluation of the proposed ML and UMAP methods. The study primarily focused on motor activity, neglecting other important features that could enhance the model’s performance. Furthermore, there was a lack of external validation and long-term monitoring, which could provide a more comprehensive understanding of the relationship between motor activity and depressive symptoms. These limitations highlight the need for future research to address these issues for improved validity and applicability of the ML and UMAP methods in depression detection and classification.

## 7. Conclusions

This paper proposes a combination of unsupervised machine learning dimensionality reduction, neural networks, and uniform manifold approximation and projection for accurate depression detection and classification. Two experiments were conducted, one with dimensional reduction and one without, to demonstrate the effectiveness of the proposed method. Various metrics, including accuracy score, F1-score, and Cohen Kappa score, were utilized to assess the model’s performance. Remarkable results were achieved, successfully distinguishing between healthy and ill instances as well as different stages of depression. The model training process was completed in fewer epochs than anticipated, thanks to the implementation of the Adam optimizer. However, while the cross-entropy loss approached zero, the validation score did not perform as well as the training score. The second experiment showed rapid convergence to a high accuracy of 0.991, aligning with the outcomes of UMAP dimensionality reduction. Furthermore, a range of machine learning classification techniques, such as the nearest neighbors, SVM, decision tree, random forest, and neural network, were employed. The highest score achieved among these algorithms was 0.727. It is worth noting that due to the limited number of samples in the Bipolar II class, especially in the testing set, the classification performance was adversely affected.

The study’s findings suggest several future research directions. These include exploring additional dimensionality reduction techniques such as t-SNE or PCA, integrating multimodal data sources for improved accuracy, conducting longitudinal studies to understand depression’s progression, validating the proposed method in clinical settings, and focusing on the interpretability and explainability of depression classification models. By pursuing these avenues, advancements can be made in the diagnosis, treatment, and support for individuals with depression.

## Figures and Tables

**Table 1:** List of past paper references with methodology used and results.

Ref.	Dataset/Parameters	Methodology/Techniques	Results
[1]	Knowledge discovery from databases (KDD).	ML, RF, Feature Selection, Data Mining, EGG, 5-fold cross-validation, major depressive disorder (MDD).	Data for each Day Stage are as follows: 06:00–11:59, 12:00–17:59, and 18:00–23:59. The maximum accuracy for these models is 98%, 88.45%, 81.65%, and 91.26%, respectively.
[2]	Motor activity database (Depresjon)	ML-based method, Bipolar disorder, unipolar depression	Specificity value of 0.89 and sensitivity value of 0.97.
[3]	Database for depression, 14 statistical parameters shown	motor activity, RF, feature extraction, and classification analysis	Specificity measures 0.920 with a sensitivity value of 0.877.
[5]	The dataset for the Motor Activity Recording had 291 depressed and 402 non-depressed situations.	ML, RF, DNN, CNN, SMOTE sampling, and piezoelectric accelerometer programming. analyses interpretability	The weighted CNN method, which accurately classified 65% of the data in the initial run, was the best-performing ML technique. Using the SMOTE oversampling method, DNN correctly categorized 82% of depressed patients and 84% of controls.
[11]	PSMU was reported by 125 pupils. Stress for 14 days, seven times each day.	PSMU, signs of depression, descriptive statistics Temporal Network and Vector-Auto Regression	A relationship between PSMU and particular depressive symptoms was established.
[16]	25 patients with major depressive disorder and 21 patients with schizophrenia were evaluated.	Supervised Convex Nonnegative Matrix Factorization, DMN, SN, CEN Network, and brain imaging research	82.6% classification accuracy was attained.
[17]	17 people underwent cognitive MRI.	Mini-Mental Status Test, Rest-Activity Rhythm (RAR) method, and English’s Mini-Mental Status Examination (3MS)	RAR fragmentation: per SD β = −0.43, 95% confidence interval (CI): −0.73, −0.14. Standard deviation β = 0.47, 95% CI: 0.14, 0.79.
[36]	Depression was present in 23 individuals. 32 individuals are devoid of depression.	Feature Extraction, Feature Selection, Genetic Algorithm, RF, and Statistical Analysis of the Montgomery–Asberg Depression Rating Scale	AUC for a motion signal can be as low as 0.647, whereas AUC for a method of feature extraction can be as high as 0.734.

**Table 2:** Dataset related to four classes.

Class	Size (N, ts)
Healthy	697, 1440
Bipolar I	117, 1440
Bipolar II	14, 1440
Unipolar	240, 1440

**Table 3:** Transformed data.

Class	Size (N, ts)
Healthy	697, 2
Bipolar I	117, 2
Bipolar II	14, 2
Unipolar	240, 2

**Table 4:** Metrics and validation score for the model.

Metrics	Validation Score
Accuracy	0.6340
F1-score	0.5694
Cohen Kappa	0.1058

**Table 5:** Metrics and score validation data.

Metrics	Score
Accuracy	0.991
F1-score	0.9887
Cohen Kappa	0.9772

**Table 6:** Comparison of other authors’ experiments and our model.

Paper	Brief Description	Accuracy	F1	Kappa
Ours	UMAP + NN	0.991	0.9887	0.9772
[36]	UMAP	0.89	/	0.773
[3]	RF	0.893	0.919	/
[5]	Linear SVM	0.727	0.727	/

## Data Availability

The dataset used in this study is available at: https://www.kaggle.com/datasets/arashnic/the-depression-dataset (accessed on 20 February 2021).
